# Effectiveness of a school-based physical activity-related injury prevention program on risk behavior and neuromotor fitness a cluster randomized controlled trial

**DOI:** 10.1186/1479-5868-7-9

**Published:** 2010-01-28

**Authors:** Dorine CM Collard, Mai JM Chinapaw, Evert ALM Verhagen, Ingrid Bakker, Willem van Mechelen

**Affiliations:** 1 EMGO Institute for Health and Care Research, Department of Public & Occupational Health, VU University Medical Center, Van der Boechorststraat 7, 1081 BT Amsterdam, The Netherlands; 2 TNO Quality of Life, Department of Physical Activity and Health, Wassenaarseweg 56, PO Box 2215, 2301 CE Leiden, The Netherlands; 3 Body@Work, Research Centre Physical Activity, Work and Health, TNO-VU University Medical Centre, Amsterdam, The Netherlands

## Abstract

**Background:**

To investigate the effects of a school-based physical activity-related injury prevention program, called 'iPlay', on risk behavior and neuromotor fitness.

**Methods:**

In this cluster randomized controlled trial 40 primary schools throughout the Netherlands were randomly assigned in an intervention (n = 20) or control group (n = 20). The study includes 2,210 children aged 10-12 years.

The iPlay-intervention takes one school year and consists of a teacher manual, informative newsletters and posters, a website, and simple exercises to be carried out during physical education classes.

Outcomes measures were self-reported injury preventing behavior, self-reported behavioral determinants (knowledge, attitude, social-influence, self-efficacy, and intention), and neuromotor fitness.

**Results:**

The iPlay-program was not able to significantly improve injury-preventing behavior. The program did significantly improve knowledge and attitude, two determinants of behavior. The effect of the intervention-program on behavior appeared to be significantly mediated by knowledge and attitude. Improved scores on attitude, social norm, self-efficacy and intention were significantly related to changes in injury preventing behavior. Furthermore, iPlay resulted in small non-significant improvements in neuromotor fitness in favor of the intervention group.

**Conclusion:**

This cluster randomized controlled trial showed that the iPlay-program did significantly improved behavioral determinants. However, this effect on knowledge and attitude was not strong enough to improve injury preventing behavior. Furthermore, the results confirm the hypothetical model that injury preventing behavior is determined by intention, attitude, social norm and self-efficacy.

**Trial number:**

ISRCTN78846684

## Introduction

The benefits of regular physical activity (PA) are widely known and include enhanced cardio respiratory fitness, increased muscular strength and endurance, and prevention of obesity [1-3]. However, participation in PA's can lead to unwanted consequences, such as injuries. Data from the period 2000-2005 revealed that in the Netherlands 1,5 million sport-related injuries are reported each year and 51% of these injuries required medical treatment [4]. The sport injury incidence in Dutch children aged 0-17 is 1.3 (95%CI:1.2-1.4) per 1000 hours sport participation [5]. PA injuries may result in pain and disability, high medical costs and school or parental work absence [6-8]. Therefore, PA-related injury prevention in children is of great relevance for public health.

School-based prevention programs are promising because of their potential to reach almost all children in the population. To our knowledge school-based injury prevention programs are lacking. Therefore, we developed a school-based injury prevention program. The aim of this program, called iPlay, was to decrease PA-related injuries by changing injury preventing behavior and neuromotor fitness [9]. A PA-related injury was defined as any injury occurring during the entire scope of PA modalities and leading at least to cessation of the current activity.

To improve injury preventing behavior, we need to change the underlying determinants [10]. The iPlay-program was based on the Attitude - Social influence - self Efficacy (ASE) model, a basic model describing determinants of health behavior. The ASE model is based on the assumption that the intention to engage in behavior is the result of the attitude, social influence and self-efficacy towards performing the specific behavior. The ASE model is based on the theory of planned behavior [11] and the social learning theory [12]. Because attitude is partly based on knowledge, improving knowledge about injury prevention was also an aim of iPlay-program.

**Figure 1:**
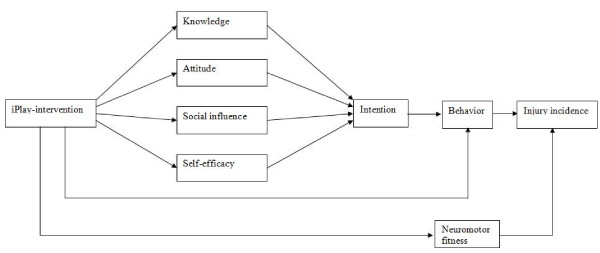
**Hypothetical model that was used to for the iPlay-program**.

In addition, iPlay also aimed to improve neuromotor fitness (e.g. flexibility, strength and balance/proprioception). Sport-specific studies suggest that improving certain dimensions of neuromotor fitness can decrease PA-related injuries [13-17]. Furthermore, in the focus groups interviews PE teachers mentioned in particular the great diversity in neuromotor fitness in children. Although this common opinion could not be supported by scientific literature, it showed that teachers believe that improvements in neuromotor fitness can decrease injury risk [9]. Additionally, low levels of neuromotor fitness may negatively affect children in their daily physical activity levels and in their health status in the long term [18,19]. Figure 1 shows the hypothetical model that was used for the iPlay-program.

We found a substantial and relevant reduction in PA-related injuries, especially in children in the low active group because of the iPlay-intervention [20]. This manuscript describes the effectiveness of the iPlay-program on injury preventing behavior, the targeted behavioral determinants (i.e. knowledge, attitude, social influence, self efficacy and intention) and neuromotor fitness. In addition, we tested whether the hypothesized behavioral determinants indeed mediated the intervention effects on behavior. Furthermore, the aim of this manuscript is to identify the mediating mechanisms targeted by the iPlay-program. Mediation analysis is useful, because it gives insight in the elements of the intervention that were successful or not.

## Methods

### Study design and participants

The effectiveness of the iPlay-program was evaluated using a cluster randomized controlled trial. From January 2006, a random sample of Dutch primary schools located in urban as well as in suburban areas were selected and invited to participate in the iPlay-study. Inclusion criteria were: being a regular primary school; providing physical education (PE) classes twice a week for 45 minutes; and willing to appoint a contact person for the duration of the study. All children from grades 5 and 6 (aged 10-12 years) were eligible to participate in the study. Before baseline measurements, we performed a stratified randomization based on geographic location of the school (urban/suburban) and professional status of the physical education teacher (certified/uncertified). Randomization took place at school level.

Parents of all participating children received a passive informed consent, involving a letter that explained the nature of the study and procedures. If parents and/or their child did not want to participate in the study they were able to indicate this. The Medical Ethics Committee of VU University Medical Centre approved the study design, protocols and informed consent procedures.

### iPlay-program

The intervention-program was developed according to the Intervention Mapping protocol [21]. The development of the iPlay-program is extensively described in another manuscript [9]. In short, the eight-month intervention-program focused on both children and parents. During one school year children received monthly newsletters aimed at improving knowledge about and attitude and self-efficacy towards the prevention of PA-related injuries. Parents received a monthly newsletter aimed at improving knowledge about and attitude towards injury prevention, and also suggesting strategies to reduce the PA injury risk in their child. Children took the parent newsletter home. Besides the newsletters, attractive posters were displayed in the classroom addressing the main intervention topics regarding PA injury prevention. The program also provided access to an informative website about injury prevention for children and parents. In addition, short 5-minute exercises were given at the beginning and end of each PE class aimed at improving muscle strength, speed, flexibility and coordination. A teachers' manual contained information about the intervention-program including time schedule, exercises, and topics of the newsletters.

### Measurements

A trained research team completed the measurements according to a standardized protocol. All children completed a questionnaire and a neuromotor fitness test at the start (September 2006) and at the end op the school year (June 2007). The questionnaire collected information on demographic variables, knowledge about injury prevention, self-reported injury preventing behavior, as well as behavioral determinants. Answers were given on a five point Likert scale varying from never (-2) to always [2] or totally not agree (-2) to totally agree [2]. All questions were positively formulated. Socio economical status (SES) was defined on the basis of the highest level of maternal education, from a parental questionnaire and ranged from 1 (no qualification) to 8 (master's degree).

#### Injury preventing behavior

A potentially modifiable risk factor for PA-related injuries in children is injury preventing behavior, i.e. not wearing appropriate protective equipment and/or footwear during PA's [22,23].

We defined PA injury preventing behavior as 1) wearing appropriate protective equipment during organized sports activities, 2) wearing appropriate protective equipment during leisure time activities, and 3) wearing appropriate footwear during PA's (i.e. organized PA's, leisure time PA's and regular PE class). Each sub-behavior was measured by one question in the questionnaire.

#### Determinants of behavior

Children completed a knowledge-test at follow-up, including nine multiple-choice questions. The total score was calculated by summing up all correct answers.

Attitude, social influence, self-efficacy, and intention were assessed at baseline and follow-up. Attitude was assessed with three questions. Social influences include social norm and modeling. Social norm was assessed with two question (e.g. 'My parents think I should wear protective materials during sports activities' Yes, totally agree...No, totally disagree)). Modeling was assessed with two questions about modeling by friends and parents. Self-efficacy was assessed with two questions relating to the child's perception of his/her ability to perform injury preventing behavior. Intention towards PA injury prevention was assessed with one question.

#### MOPER fitness test

Children performed 7 test items of the MOPER fitness test [24] during one PE class (bent arm hang test to measure upper body strength, 10 times 5-m run test to measure running speed and agility, plate tapping test to measure eye-hand coordination and arm speed, leg lift test to measure trunk/leg strength, sit and reach test to measure trunk flexibility, arm pull test to measure static arm strength and standing high jump test to measure explosive leg strength). Validity and reliability of the MOPER fitness test have been shown to be acceptable [25]. For logistic reasons and since iPlay did not specifically focus on improving aerobic endurance we decided to exclude the 6 minutes endurance run. In addition to the 7 test items, children performed the flamingo balance test to measure general balance [26]. To be able to complete all tests during one PE class, we shortened the flamingo balance test to 30 seconds, instead of one minute as the original flamingo balance test protocol indicates.

All test items were performed barefoot to rule out the effect of footwear on the test results. In addition, children were encouraged to perform all test elements as good as possible.

#### Anthropometrics

Body height was measured in meters (m), with a portable stadiometer (Seca 214, Leicester Height Measure; Seca GmbH & Co, Hamburg, Germany) with the subject standing straight, with the heels together and looking straight ahead. Body weight was measured in kilogram (kg), with a digital scale (Seca 770; Seca GmbH & Co, Hamburg, Germany). BMI was calculated by the weight in kilograms divided by height in meters squared (kg/m^2^).

### Statistical analyses

To compare the intervention and control group at baseline, we used the Pearson Chi-Square test (gender, SES and BMIclass) and the independent samples-t-test (age and BMI).

To test the hypothetical iPlay-model (figure 1) a mediation analyses was performed using single and multiple two-level linear regression models (child and school), accounting for within-school cluster effects. The single mediator model reflects the intervention effect on the outcome measure through each mediating variable. A multiple mediator model was used to assess the independent contribution of each single mediator because the mediated effects of the potential mediators may overlap.

First, we calculated the effect of the iPlay-program on the behavioral outcomes (τ). Next, we estimated the effect of the intervention on behavioral determinants (α-coefficients). Then we estimated the independent effect of changes in determinants of behavior on changes in behavior (β coefficient). Change scores are the post-intervention scores, adjusted for the pre-intervention scores and therefore represent change adjusted for baseline values. We estimated the magnitude of the mediated effect over time by computing the product of the α- and β-coefficients. Finally, the statistical significance of the mediating effect was calculated by dividing the mediated effect (α* β) by its standard error. Social modeling was not included in the analysis due to too much missing values.

Multi-level linear regression analysis was used to analyze between-group differences in neuromotor fitness test scores. Schools were used as a cluster level. All analyses were performed according to the intention-to-treat-principle using MLWin 2.15 adjusting for baseline values, SES, BMI and gender.

## Results

### Participants

**Figure 2:**
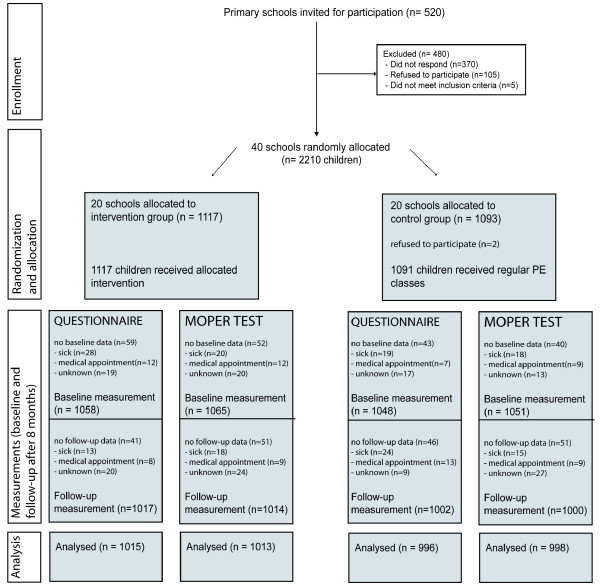
**Flow chart of schools and participants**.

A total of 2,210 children from 40 primary schools throughout the Netherlands participated in the study. Figure 2 outlines the complete flow of participants from recruitment through the last follow-up contact. Reasons for not completing the questionnaire or the MOPER test were mostly school absence due to illness or having a medical appointment. Eight questionnaires and three MOPER fitness test score forms were completed inappropriate and therefore excluded from analyses. Eventually, questionnaire data from 1,015 children in the intervention group and 996 children in the control group were analyzed. Furthermore, MOPER fitness test data from 1,013 children in the intervention group and 998 children in the control group were analyzed.

The mean age of the children was 10.7 ± 0.8 years. Intervention and control group were similar regarding age and gender. BMI in the control group (18.1 ± 3.1 kg/m^2^) was significantly higher than in the intervention group (17.7 ± 2.7 kg/m^2^). In addition, the intervention group included significantly more children from a low SES.

Table 1 shows the baseline and follow-up values for self-reported behavior and determinants of behavior towards wearing protective equipment during organized sports activities and leisure time activities and wearing appropriate footwear during PA's.

**Table 1:** Baseline en follow-up behavior and determinants of behavior in intervention and control group.

	Intervention group			Control group		
	**N**	**Baseline** **Mean ± SD**	**Follow-up** **Mean ± SD**	**N**	**Baseline** **Mean ± SD**	**Follow-up** **Mean ± SD**

**Wearing protective equipment during organized sports activities**						

Behavior (-2; 2)^a^	531	1.7 (0.8)	1.7 (0.8)	455	1.6 (1.0)	1.6 (0.9)

Attitude (-2; 2)^a^	564	1.6 (0.5)	1.6 (0.5)	480	1.6 (0.5)	1.6 (0.5)

Social norm (-2; 2)^a^	571	1.1 (0.9)	1.0 (0.8)	484	1.1 (0.9)	1.0 (0.9)

Self efficacy (-2; 2)^a^	552	1.7 (0.5)	1.7 (0.5)	469	1.7 (0.5)	1.7 (0.5)

Intention (-2; 2)^a^	570	1.4 (1.2)	1.3 (1.3)	468	1.4 (1.2)	1.3 (1.3)

**Wearing protective equipment during leisure time activities**						

Behavior (-2; 2)^a^	605	-0.5 (1.4)	-0.6 (1.4)	516	-0.5 (1.5)	-0.5 (1.5)

Attitude (-2; 2)^a^	605	0.6 (1.0)	0.5 (0.9)	521	0.7 (0.9)	0.6 (0.9)

Social norm (-2; 2)^a^	607	0.1 (1.0)	0.1 (1.1)	525	0.2 (1.0)	0.1 (1.0)

Self efficacy (-2; 2)^a^	598	0.9 (1.0)	0.8 (1.1)	520	1.0 (1.0)	0.9 (1.0)

Intention (-2; 2)^a^	605	-0.0 (1.5)	-0.0 (1.5)	526	0.1 (1.6)	-0.0 (1.6)

**Wearing appropriate footwear during organized, leisure time PA's and PE classes.**						

Behavior (-2; 2)^a^	799	1.1 (0.9)	1.2 (0.9)	712	0.9 (1.0)	1.1 (1.0)

Attitude (-2; 2)^a^	993	0.9 (0.8)	1.0 (0.7)	983	0.8 (0.7)	0.8 (0.7)

Social norm (-2; 2)^a^	995	0.6 (0.9)	0.6 (0.9)	982	0.6 (1.0)	0.5 (1.0)

Self efficacy (-2; 2)^a^	980	1.5 (0.6)	1.5 (0.7)	956	1.4 (0.6)	1.4 (0.6)

Intention (-2; 2)^a^	949	1.2 (1.0)	0.6 (0.7)	939	1.1 (1.0)	0.6 (0.7)

^a ^a higher score on the Likert-scale is more favorable.

### Intervention effects on injury preventing behaviors (τ)

The second column of table 2, 3 and 4 represents the iPlay-intervention effects on the three injury preventing behaviors. The intervention did not significantly affect the behavior of the children towards wearing protective equipment during organized sports activities (τ = 0.05 (95%CI = -0.04 - 0.14)), wearing protective equipment during leisure time activities (τ = -0.01 (95%CI = -0.21 - 0.19)) or wearing appropriate footwear during PA's (τ = 0.07 (95%CI = -0.13 - 0.27).

**Table 2:** Wearing protective equipment during organized sports activities

	Effect on behavior (τ) (95%CI)	Effect on determinants of behavior (α) (95%CI)	Effect of determinants of behavior on behavior (β) (95%CI)		Mediated effect (α* β) (95%CI)	
			**Single-mediator ** **model**	**Multiple-mediator ** **model**	**Single-mediator ** **model**	**Multiple-mediator ** **model**

**Protective equipment ** **organized sports**	0.05 (-0.04-0.14)					

Knowledge (-2; 2)^a^		0.49 * (0.20-0.78)	0.05 * (0.01-0.08)	0.03 (-0.00-0.06)	0.02 * (0.02-0.03)	0.02 * (0.01-0.02)

Attitude (-2; 2)^a^		-0.01 (-0.08-0.06)	0.28 * (0.18-0.38)	0.15 * (0.02-0.27)	-0.00 (-0.02-0.02)	-0.00 (-0.01-0.01)

Social norm (-2; 2)^a^		-0.01 (-0.11-0.09)	0.10 * (0.04-0.16)	0.06 (-0.01-0.13)	-0.00 (-0.01-0.01)	0.00 (-0.01-0.01)

Self efficacy (-2; 2)^a^		-0.01 (-0.07-0.05)	0.10 (-0.01-0.21)	0.04 (-0.09-0.17)	-0.00 (-0.01-0.01)	0.00 (-0.00-0.00)

Intention (-2; 2)^a^		-0.14 (-0.40-0.13)	0.09 * (0.06-0.13)	0.09 * (0.04-0.13)	-0.01 (-0.04-0.01)	-0.01 (-0.03-0.01)

^a ^a higher score on the Likert-scale is more favorable.

* significant effect (p < 0.05)

**Table 3:** Wearing protective equipment during leisure time activities

	Effect on behavior (τ) (95%CI)	Effect on determinants of behavior (α) (95%CI)	Effect of determinants of behavior on behavior (β) (95%CI)		Mediated effect (α* β) (95%CI)	
			**Single-mediator ** **model**	**Multiple-mediator ** **model**	**Single-mediator ** **model**	**Multiple-mediator ** **model**

**Protective equipment leisure ** **time activities**	-0.01 (-0.21-0.19)					

Knowledge (-2; 2)^a^		0.49 * (0.20-0.78)	0.10 * (0.03-0.16)	0.03 (-0.02-0.08)	0.05 * (0.04-0.06)	0.01 * (0.01-0.02)

Attitude (-2; 2)^a^		-0.04 (-0.16-0.09)	0.65 * (0.56-0.75)	0.26 * (0.14-0.38)	-0.02 (-0.10-0.06)	-0.01 (-0.04-0.02)

Social norm (-2; 2)^a^		-0.02 (-0.14-0.11)	0.57 * (0.49-0.66)	0.25 * (0.15-0.34)	-0.01 (-0.08-0.06)	-0.00 (-0.03-0.03)

Self efficacy (-2; 2)^a^		-0.15 * (-0.27- -0.03)	0.36 * (0.28-0.44)	0.11 * (0.03-0.19)	-0.06 * (-0.10- -0.01)	-0.02 (-0.04-0.00)

Intention (-2; 2)^a^		0.12 (-0.08-0.32)	0.44 * (0.39-0.50)	0.27 * (0.21-0.34)	0.05 (-0.04-0.14)	0.03 (-0.02-0.09)

^a ^a higher score on the Likert-scale is more favorable.

* significant effect (p < 0.05)

**Table 4:** Wearing appropriate footwear during organized, leisure time activities and PE classes.

	Effect on behavior (τ) (95%CI)	Effect on determinants of behavior (α) (95%CI)	Effect of determinants of behavior on behavior (β) (95%CI)		Mediated effect (α* β) (95%CI)	
			**Single-mediator ** **model**	**Multiple-mediator ** **model**	**Single-mediator ** **model**	**Multiple-mediator ** **model**

**Appropriate footwear during ** **physical activities**	0.07 (-0.13-0.27)					

Knowledge (-2; 2)^a^		0.49 * (0.20-0.78)	0.03 (-0.01-0.06)	0.00 (-0.03-0.03)	0.01 * (0.01-0.02)	0.00 (-0.00-0.00)

Attitude (-2; 2)^a^		0.10 * (0.00-0.20)	0.28 * (0.21-0.35)	0.08 * (0.00-0.16)	0.03 * (0.00-0.06)	0.01 (-0.00-0.02)

Social norm (-2; 2)^a^		0.13 (-0.02-0.28)	0.21 * (0.16-0.26)	0.01 * (0.04-0.16)	0.03 (-0.00-0.06)	0.01 (-0.00-0.03)

Self efficacy (-2; 2)^a^		0.04 (-0.04-0.12)	0.55 * (0.48-0.63)	0.46 * (0.38-0.55)	0.02 (-0.03-0.07)	0.02 (-0.02-0.06)

Intention (-2; 2)^a^		0.06 (-0.01-0.14)	0.16 * (0.10-0.23)	0.05 (-0.01-0.12)	0.01 (-0.00-0.02)	0.00 (-0.00-0.01)

^a ^a higher score on the Likert-scale is more favorable.

* significant effect (p < 0.05)

### Intervention effects on behavioral determinants (α-coefficients)

The third column of table 2, 3 and 4 represents the intervention effects on behavioral determinants i.e. knowledge, attitude, social norm, self-efficacy and intention.

The iPlay-program significantly improved knowledge about injury prevention (α = 0.49 (95%CI = 0.20 - 0.78)). In addition, the iPlay-program also significantly improved attitude towards wearing appropriate footwear during PA's (α = 0.10 (95%CI = 0.00 - 0.20)) (table 4). Furthermore, we found a significant negative effect of the iPlay-program on self-efficacy towards wearing protective equipment during leisure time activities (α = -0.15 (95%CI = -0.27 - -0.03)) (table 3). The intervention did not significantly affect the other determinants.

### Determinant effects on behaviors (β-coefficients)

Next we checked whether the changes in the determinants were associated with changes in the three injury preventing behaviors (β-coefficients in column 4 and 5 of table 2, 3 and 4).

Improved scores on knowledge (β = 0.05 (95%CI = 0.01 - 0.08)), attitude (β = 0.28 (95%CI = 0.18 - 0.38)), social norm (β = 0.10 (95%CI = 0.04 - 0.16)) and intention (β = 0.09 (95%CI = 0.06 - 0.13)) were significantly related to wearing more often protective equipment during organized sport activities (table 2, column 4).

Improved scores on knowledge (β = 0.10 (95%CI = 0.03 - 0.16)), attitude (β = 0.65 (95%CI = 0.56 - 0.75)), social norm (β = 0.57 (95%CI = 0.49 - 0.66)), self-efficacy (β = 0.36 (95%CI = 0.28-0.44), and intention (β = 0.44 (95%CI = 0.39 - 0.50)) were significantly related to wearing more often protective equipment during leisure time activities (table 3, column 4).

Improved scores on attitude (β = 0.28 (95%CI = 0.21 - 0.35)), social norm (β = 0.21 (95%CI = 0.16 - 0.26)), self-efficacy (β = 0.55 (95%CI = 0.48-0.63), and intention (β = 0.16 (95%CI = 0.10 - 0.23)) were significantly related to wearing more often appropriate footwear during PA's (table 4, column 4).

Since the intervention targeted multiple determinants simultaneously, the effects of the determinants on injury preventing behavior change were also assessed in a multiple-mediator model to account for multicollinearity (table 2, 3 and 4, column 5).

Improved scores on attitude (β = 0.15 (95%CI = 0.02 - 0.27)), intention (β = 0.09 (95%CI = 0.04 - 0.13)) and knowledge (β = 0.03 (95%CI = -0.00 - 0.06) were significantly related to wearing more often protective equipment during organized sport activities, although this latter association was borderline significant (table 2, column 5).

Improved scores on attitude (β = 0.26 (95%CI = 0.14 - 0.38)), social norm (β = 0.25 (95%CI = 0.15 - 0.34)), self-efficacy (β = 0.11 (95%CI = 0.03-0.19), and intention (β = 0.27 (95%CI = 0.21 - 0.34)) were significantly related to wearing more often protective equipment during leisure time activities (table 3, column 5).

Improved scores on attitude (β = 0.08 (95%CI = 0.00 - 0.16)), social norm (β = 0.01 (95%CI = 0.04 - 0.16)), self-efficacy (β = 0.46 (95%CI = 0.38-0.55) were significantly related to wearing more often appropriate footwear during PA's (table 4, column 5).

### Mediation (α*β-coefficients)

Respectively, column 6 and 7 of table 2, 3 and 4 represent the single and multiple mediated effects.

The single-mediator model showed that the intervention effect of the iPlay-program on changes in wearing protective equipment during organized sport activities was mediated by knowledge (αβ = 0.02 (95%CI = 0.02-0.03)). Thus, the improvement in knowledge partly explained the change in wearing protective equipment during organized sport activities (table 2, column 6). However, the effects were small. The intervention effect on wearing protective equipment during leisure time activities was also mediated by knowledge (αβ = 0.05 (95%CI = 0.04-0.06)). The single-mediator model revealed also a statistically significant suppression effect of self-efficacy on changes in wearing protective equipment during leisure time activities (αβ = -0.06 (95%CI = -0.10 - -0.01) (table 3, column 6). Unfortunately, the iPlay-program had a negative effect on self-efficacy for wearing protective equipment during leisure time activities. In the multiple-mediator model, this suppression effect was no longer significant (table 3, column 7).

The intervention effect of the iPlay-program on wearing appropriate footwear during PA's was mediated by knowledge (αβ = 0.01 (95%CI = 0.01 - 0.02)) and attitude (αβ = 0.03 (95%CI = 0.00 - 0.06) (table 4, column 6). No significant mediated effects were found in the multiple-mediator model.

### Motor fitness

Table 5 and 6 present the results regarding the MOPER fitness test items for boys and girls, respectively. Separate analyses were conducted for boys and girls, as gender was found to be an effect modifier. There was a trend towards improvement on almost all MOPER fitness test items in boys and girls in favor of the intervention group. In boys, no significant intervention effect on the MOPER fitness test items was found. In girls, a significant beneficial intervention effect on the 10 × 5 m run was found.

**Table 5:** Intervention effects on MOPER fitness test scores for boys.

BOYS	Intervention group		Control group		**Adjusted **^a ^**difference between groups**
	**Baseline** **(mean ± SD)**	**Follow-up** **(mean ± SD)**	**Baseline** **(mean ± SD)**	**Follow-up** **(mean ± SD)**	**β** **(95%CI)**

**Bent arm hang (sec)** Median (25-75 IQR)	10 (4 - 20)	10 (4 - 20)	8 (3 - 18)	10 (4 - 21)	0.39 † (-1.35-2.14)

**10 × 5 run** **(sec)**	19.5 ± 1.5	19.1 ± 1.5	19.5 ± 1.6	19.2 ± 1.5	-0.09 † (-1.35-0.18)

**Leg lift** **(sec)**	16.6 ± 1.3	17.4 ± 5.9	17.6 ± 1.4	17.2 ± 1.4	-0.40 † (-1.62-0.81)

**Plate tapping** **(sec)**	15.1 ± 2.0	13.5 ± 1.6	15.0 ± 1.9	13.7 ± 1.8	-0.24 † (-0.53-0.06)

**Sit and reach** **(cm)**	26 ± 6	26 ± 7	26 ± 7	26 ± 7	0.22 † (-0.39-0.83)

**Arm pull** **(kg/kg weight)**	68 ± 7	73 ± 9	70 ± 3	73 ± 8	-1.21 (-7.42-5.00)

**Standing high jump (cm)**	38 ± 6	39 ± 7	38 ± 7	39 ± 7	-0.12 (-1.20-0.97)

**Flamingo (attempts)**	8 ± 3	8 ± 3	8 ± 3	8 ± 3	-0.17 † (-0.69-0.35)

^a ^adjusted for baseline value, SES and BMI.

† changes in favor of the intervention group.

**Table 6:** Intervention effects on MOPER fitness test scores for girls.

GIRLS	Intervention group		Control group		**Adjusted **^a ^**difference between groups**
	**Baseline** **(mean ± SD)**	**Follow-up** **(mean ± SD)**	**Baseline** **(mean ± SD)**	**Follow-up** **(mean ± SD)**	**β** **(95%CI)**

**Bent arm hang (sec)** Median (25-75 IQR)	6 (3 - 13)	8 (3 - 15)	6 (2 - 13)	6 (2 - 12)	2.08 † (-0.34-3.83)

**10 × 5 run** **(sec)**	20.0 ± 1.5	19.3 ± 1.6	20.0 ± 1.6	19.7 ± 1.5	-0.33 † * (-0.50- -0.16)

**Leg lift** **(sec)**	16.7 ± 1.3	16.4 ± 1.3	17.3 ± 1.4	16.9 ± 1.4	-0.80 † (-1.83-0.23)

**Plate tapping** **(sec)**	14.7 ± 1.8	13.4 ± 1.8	14.8 ± 1.9	13.5 ± 1.7	-0.17 † (-0.47-0.14)

**Sit and reach** **(cm)**	30 ± 6	30 ± 7	30 ± 6	30 ± 7	0.47 † (-0.39-1.32)

**Arm pull** **(kg/kg weight)**	61 ± 4	67 ± 5	62 ± 2	64 ± 6	3.44 † (-1.64-8.51)

**Standing high jump (cm)**	37 ± 6	38 ± 7	36 ± 7	36 ± 7	0.82 † (-0.47-2.10)

**Flamingo (attempts)**	8 ± 3	7 ± 3	8 ± 3	7 ± 3	0.09 (-0.27-0.46)

^a ^adjusted for baseline value, SES and BMI.

† changes in favor of the intervention group.

* significant differences between intervention and control group.

## Discussion

This manuscript describes the effects of the iPlay-program on injury preventing behavior, the targeted behavioral determinants and neuromotor fitness. Furthermore, we examined the underlying hypothetical model.

The iPlay-program did not improve behavior towards wearing protective equipment and appropriate footwear during PA's despite the fact that the iPlay-program significantly changed knowledge about injury prevention and attitude towards wearing appropriate footwear during PA's. The negative effect on self-efficacy towards wearing protective equipment during leisure time activities that was found can be possibly explained by the fact that children perceived more barriers after the intervention decreasing their self-efficacy [10].

Several explanations can be suggested for the minimal effects of the iPlay-program on behavior and determinants of behavior. First, the self-reported measurements might not have been sufficiently sensitive to detect changes in behavior and determinants of behavior. Injury preventing behavior and the determinants were measured using an invalidated and self-reported questionnaire. Self-report measures have numerous limitations such as social desirability (integration bias) and recall bias. In addition, constructs such as intention and behavior were measured with only one question. Possibly, having one question as an index of behavior and intention is not adequate enough.

A second explanation can be that the iPlay-program was not adequately implemented, which has led to a lack of impact on behavior and its determinants. However, a positive effect on knowledge about injury prevention suggests that the program was at least partly implemented.

A third explanation for the lack of effect on wearing protective equipment and its determinants is possibly explained by the fact that at baseline almost all children indicated that they were already wearing protective equipment during organized sports activities. Improvement of this particular behavior was therefore difficult.

A last possible explanation for the lack of effect of the intervention could be that the intervention methods or strategies used in this intervention (active learning, providing cues and scenario-based risk information and active processing of information) were not effective. Possibly, other methods or strategies to improve attitude, socials norm, self-efficacy and intention should be used.

A second overall conclusion based on the results presented in this manuscript is that improved scores on knowledge, attitude, social norm, self-efficacy and intention were significantly related to changes in injury preventing behaviors. These results confirm our hypothetical model that behavior is determined by intention, attitude, social norm and self-efficacy [27,28].

Unfortunately, the iPlay-program was not capable to improve social norm, self-efficacy and intention. Improvements of the iPlay-program should focus on strategies to increase scores on those determinants.

A third conclusion of this study is that the intervention effect on injury preventing behavior was mediated by changes in knowledge and attitude. However, the intervention effect was too small to lead to actual behavior change.

Finally we can conclude that almost all MOPER fitness test items showed small improvements in favor of the intervention group. Although not significant, the effects of the iPlay-program appear promising.

### Comparison with previous research

To our knowledge, the iPlay-program is the first school-based injury prevention program for children aged 10-12 years aimed at decreasing PA-related injuries by improving injury preventing behavior and neuromotor fitness. Backx [29] conducted a school-based intervention aimed at preventing PA injuries in adolescents aged 12-20 years. This was a smaller uncontrolled study including 471 adolescents from one secondary school that showed positive effects on knowledge and attitude. Our study, including more than 2,200 primary school children, showed consistent findings regarding knowledge and attitude.

### Strengths of the iPlay-study

The strength of our study is the large sample size. The iPlay-program has been evaluated in a randomized controlled trial including 40 primary schools with more than 2,200 children. During the study high follow-up rates were achieved in both the intervention and control group. The study population - children from different primary schools in urban and suburban areas throughout the Netherlands - was a representation of the Dutch population. Furthermore, the iPlay-program was developed using the Intervention Mapping protocol. The development was performed in collaboration with users of the intervention - teachers and school boards - and the target population - i.e. children. The iPlay-program is designed to be a workable and time-efficient program that fits into the regular school curriculum.

A limitation of the study is that - besides that there were no valid measures available for our behavioral measures - the participants and research-assistants were not blinded. Blinding of participants and research-assistants is important to prevent bias but difficult in community based studies.

## Conclusion

The iPlay-intervention aimed at the prevention of PA-related injuries in primary school children by improving injury preventing behavior and neuromotor fitness. This manuscript showed that the iPlay-program was not able to significantly improve injury preventing behavior. The effect of the intervention-program on behavior appeared to be significantly mediated by knowledge and attitude. However, the effect of the iPlay-program on knowledge and attitude was not strong enough to change injury preventing behavior. Furthermore, we found that changes in attitude, social norm, self-efficacy and intention were significantly related to changes in injury preventing behavior. Finally, iPlay resulted in small non-significant improvements in neuromotor fitness in favor of the intervention group.

## Declaration of competing interests

The authors declare that they have no competing interests.

## Authors' contributions

DC, MC, EV, IB and WvM contributed to the study design. DC was responsible for developing, testing, finalizing the intervention and data collection and entry. DC and MC conducted the data analyses and DC wrote the first draft of the manuscript. All authors (DC, MC, EV, IB and WvM) contributed to the final manuscript by reading and correcting the draft versions.

## References

[B1] Adirim TA Cheng TL Overview of injuries in the youngathlete Sports Medicine 2003 33 1 75 81 10.2165/00007256-200333010-00006 12477379

[B2] Ekblom B Astrand PO Role of physical activity on health in children and adolescents Acta Paediatrica 2000 89 7 762 4 10.1080/080352500750043576 10943950

[B3] Hallal PC Victora CG Azevedo MR Wells JC Adolescent physical activity and health: a systematic review Sports Med 2006 36 12 1019 30 10.2165/00007256-200636120-00003 17123326

[B4] Hildebrandt VH Ooijendijk WTM Hopman-Rock M Trendrapport: bewegen en gezondheid 2006-2007 2008

[B5] Hildebrandt VH Ooijendijk WTM Hopman-Rock M Trendrapport: bewegen en gezondheid 2004-2005 2007 Leiden: TNO Kwaliteit van Leven

[B6] Abernethy L MacAuley D Impact of school sports injury Br J Sports Med 2003 37 4 354 5 10.1136/bjsm.37.4.354 12893724 PMC1724674

[B7] de Loes M Dahlstedt LJ Thomee R A 7-year study on risks and costs of knee injuries in male and female youth participants in 12 sports Scand J Med Sci Sports 2000 10 2 90 7 10.1034/j.1600-0838.2000.010002090.x 10755279

[B8] MacKay M Scanlan A Olsen L Sports and recreation injury prevention strategies: systematic review and best practices: executive summary 2008 Vancouver, BC: BC Injury Research andPrevention Unit

[B9] Collard DC Chinapaw MJ van Mechelen W Verhagen EA Design of the iPlay Study: Systematic Development of a Physical Activity Injury Prevention Programme for Primary School Children Sports Med 2009 39 11 889 901 10.2165/11317880-000000000-00000 19827858

[B10] Damoiseax V Molen HT van der Kok G Gezondheidsvoorlichting en gedragsverandering 1993 van Gorcum, Assen

[B11] Fishbein M Ajzen I Belief, Attitude, Intention and Behavior: an introduction to theory and research 1975 Wiley, New York

[B12] Bandura A Social foundations of thought and action: a social cognitive theory 1986 Englewood Cliffs, NY:Prentice Hall

[B13] Emery CA Cassidy D Klassen TP The effectiveness of a proprioceptive balance-training program in healthy adolescents; a cluster randomized controlled trial Am J Epidemiol 2004 159 Ref Type: Abstract 10.1503/cmaj.1040805 PMC552888 15767608

[B14] Heidt RS Jr Sweeterman LM Carlonas RL Traub JA Tekulve FX Avoidance of soccer injuries with preseason conditioning Am J Sports Med 2000 28 5 659 62 11032220 10.1177/03635465000280050601

[B15] Hewett TE Lindenfeld TN Riccobene JV Noyes FR The effect of neuromuscular training on the incidence of knee injury in female athletes A prospective study. Am J Sports Med 1999 27 6 699 706 10.1177/03635465990270060301 10569353

[B16] Junge A Rosch D Peterson L Graf-Baumann T Dvorak J Prevention of soccer injuries: a prospective intervention study in youth amateur players Am J Sports Med 2002 30 5 652 9 12238997 10.1177/03635465020300050401

[B17] Olsen OE Myklebust G Engebretsen L Holme I Bahr R Exercises to prevent lower limb injuries in youth sports: cluster randomised controlled trial BMJ 2005 330 7489 449 10.1136/bmj.38330.632801.8F 15699058 PMC549653

[B18] Tomkinson GR Hamlin MJ Olds TS Secular Changes in Anaerobic Test Performance in Australasian Children and Adolescents Pediatric Exercise Science 2006 18 3

[B19] Tomkinson GR Global changes in anaerobic fitness test performance of children and adolescents (1958-2003) Scand J Med Sci Sports 2007 17 5 497 507 17181769 10.1111/j.1600-0838.2006.00569.x

[B20] Collard DC Verhagen EA Chinapaw MJ Knol DL van Mechelen W Effectiveness of a school-based physical activity injury prevention program: a cluster randomized controlled trial Arch Pediatr Adolesc Med 2010 20124143 10.1001/archpediatrics.2009.256

[B21] Bartholomew LK Parcel GS Kok G Gottlieb N Planning health promotion programs, an intervention mapping approach Jossey-Bass 2006

[B22] Emery CA Risk factors for injury in child and adolescent sport: a systematic review of the literature Clin J Sport Med 2003 13 4 256 68 10.1097/00042752-200307000-00011 12855930

[B23] van Mechelen W Hlobil H Kemper HC Incidence, severity, aetiology and prevention of sports injuries. A review of concepts Sports Med 1992 14 2 82 99 10.2165/00007256-199214020-00002 1509229

[B24] Kemper HC Verschuur R Bovend'eerdt J The MOPER Fitness test: A practical approach to motor performance tests in physical education in the Netherlands S Afr J Respir Sport Phys Educ Recreat 1979 2 81 93

[B25] Leyten C de MOPER fitheidstest: onderzoeksverslag 9 t/m 11 jarigen De Vrieseborch 1982

[B26] Adam C Klissouras V Ravazzolo M Renson R Tuxworth. Handbook for the EUROFIT test of Physical Fitness Council of Europe committee for the development of sport 1988

[B27] de Vries H Dijkstra M Kuhlman P Self-efficacy: the third factor besides attitude and subjective norm as apredictor of behavioural intentions Health education research 1988 3 273 82 10.1093/her/3.3.273

[B28] Kok G de Vries H Mudde A Stretcher V Planned health education and role of self-efficacy: Dutch Research Health education research 1991 6 231 8 10.1093/her/6.2.231

[B29] Backx FJG Sports injuries in youth; etiology and prevention (thesis) Janus Jongbloed Research Center on Sports and Health 1991 Rijksuniversiteit Utrecht, The Netherlands

